# Morphological and physiological responses of *Heteropogon contortus* to drought stress in a dry-hot valley

**DOI:** 10.1186/s40529-016-0131-0

**Published:** 2016-08-08

**Authors:** Xue-mei Wang, Li Zhao, Bang-guo Yan, Liang-tao Shi, Gang-cai Liu, Yu-xiao He

**Affiliations:** 1 Key Laboratory of Mountain Surface Processes and Ecological Regulation, Institute of Mountain Hazards and Environment, Chinese Academy of Sciences and Ministry of Water Resources, Chengdu, 610041 China; 2 University of Chinese Academy of Sciences, Beijing, 100049 China; 3 College of Environment and Resource Science, Southwest University of Science and Technology, Mianyang, 621010 China; 4 Institute of Tropical Eco-agricultural Sciences, Yunnan Academy of Agricultural Sciences, Yuanmou, 651300 Yunnan Province China; 5 Institute of Resources and Environment, He’nan Polytechnic University, Jiaozuo, 454000 He’nan China

**Keywords:** *Heteropogon contortus*, Drought stress, Biomass allocation, Leaf water potential, Relative water content, Chlorophyll fluorescence

## Abstract

**Background:**

*Heteropogon contortus* is a valuable pasture species that is widely used for vegetation restoration in dry-hot valleys of China. However, to date, its morphological and physiological responses to drought, and the underlying mechanisms are not well understood. This study was aimed to investigate the morphological and physiological changes of *H. contortus* under drought stress during the dry-hot season. *Heteropogon contortus* was planted in pots and subjected to four levels of soil water treatments: above 85 % (control), 70–75 % (light stress), 55–60 % (moderate stress) or 35–40 % (severe stress) of field capacity.

**Results:**

Within the total stress period (0–29 days), *H. contortus* grew rapidly in the light stress, whereas severe stress had a negative impact on growth. Aboveground biomass decreased together with increasing drought stress, whereas root biomass increased. Consequently, the root/shoot ratio of the severe stress treatment increased by 80 % compared to that of the control treatment. The ratio of bound water/free water (BW/FW) was the most sensitive parameter to drought and showed a value under severe stress that was 152.83 % more than that in the control treatment. Although leaf water potential (LWP) and leaf relative water content (RWC) decreased with progressive water stress, *H. contortus* managed to maintain a relatively high RWC (nearly 70 %) in the severe stress condition. We also detected a significant reduction (below 0.6) in the ratio of variable fluorescence/maximum fluorescence (Fv/Fm) in the severe stress treatment.

**Conclusions:**

Our results show that *H. contortus* adapts to drought mainly by avoidance mechanisms, and its morphological and physiological characteristics are inhibited under severe stress, but can recover at a certain time after re-watering. These findings might help limited water resources to be fully used for vegetation management in the studied region.

## Background

According to the IPCC (2014), climate change in most dry subtropical regions will reduce renewable water resources greatly. Therefore, water shortage will become more serious in the near future and research into plant response to water shortage is vital for improving agricultural management practices and predicting the fate of natural vegetation (Chaves et al. 2003). Both morphological and physiological changes occur when plants are subjected to water stress or drought (Pugnaire et al. 1996; Xu et al. 2006; Gazanchian et al. 2007). These morphological and physiological responses show an array of different survival strategies under drought (Galmes et al. 2007), including drought avoidance, drought tolerance, drought escape, and drought recovery (Fang and Xiong 2015).

Morphological responses to drought stress have been widely studied, with a focus on seedling growth, and biomass accumulation and partition (Zhang et al. 2004; Lei et al. 2006; Anjum et al. 2011). Plant growth is greatly inhibited under drought conditions (Rodiyati et al. 2005). Compared with the well-watered treatment, drought treatments reduce both shoot and root biomass; the reduction in shoot biomass is more pronounced than that in root biomass, causing an increase in the root/shoot (R/S) ratio (Fernandez and Reynolds 2000; Blum 2005; Haffani et al. 2014). However, Bahrani et al. (2010) showed that no forage grasses possessed a significantly different R/S ratio than the control. Furthermore, Guenni et al. (2002) found that drought treatment decreased the R/S ratio. Therefore, the pattern of biomass allocation might differ between tropical grass species.

Physiological parameters are more sensitive than morphological parameters in distinguishing the control and drought treatments (Liu et al. 2015). In general, water stress reduces the relative water content (RWC) and leaf water potential (LWP) (Guenni et al. 2004). According to LWP and RWC, desiccation-tolerant plants can be divided into those that show dehydration avoidance (a high RWC maintained at a low LWP) or dehydration tolerance (a low RWC and low LWP) (Kozlowski and Pallardy 2002). In addition, water stress-induced stomatal closure depletes intercellular CO_2_, leading to photoinhibition (Sayed 2003). Despite improvements in technology and the evolution of modulated systems, changes in the maximum quantum efficiency (Fv/Fm) and initial fluorescence (F_0_) are still accepted and widely used as a reliable diagnosis of photoinhibition (Maxwell and Johnson 2000). A reduction in Fv/Fm might represent either a reversible photoprotective down-regulation or an irreversible inactivation of PSII; it is important to distinguish between increases in F_0_ and decreases in Fv (Araus et al. 1998).

Dry-hot valleys represent a type of semi-arid and arid zone in southwest China, covering a total area of approximately 4840 km^2^ (Xiong et al. 2005). The primary vegetation in dry-hot valleys belongs to the polyclimax type, including river valley monsoon forest and thorn forest (Liu et al. 2010). However, due to the unique climate in this area, especially the water and temperature conditions, the dominant secondary vegetation types in dry-hot valley are savanna and semi-savanna (Zhang et al. 2010). The ecosystem is fragile and difficult to recover from the degraded ecosystem. The key to restoring the degraded system is improving the soil moisture conditions; however, water is the main limiting factor for the development of the area.

*Heteropogon contortus* is a tropical perennial C_4_ grass with a native distribution that includes southern Africa, southern Asia, northern Australia and Oceania, and is one of the dominant species in the soil seed bank and aboveground vegetation in dry-hot valleys (Luo and Wang 2006). It is a valuable species of vegetation restoration, and is tolerant of low resources, such as nutrients and water (Goergen and Daehler 2001, 2002). Van den Berg and Zeng (2006) studied the effect of drought on some morphological characteristics of *H. contortus* seedlings. Williams and Black (1994) compared the drought responses of *H. contortus* with *Pennisetum setaceum*, an exotic species; however, the study mainly clarified the successful invasion of *P. setaceum*, and the physiological mechanism of drought resistance of *H. contortus* remains unclear. In summary, little is known about the effect of drought on the morphological and physiological traits of *H. contortus*. Therefore, the objectives of this study were to: (1) test how *H. contortus* adapts to water stress by belowground and aboveground biomass allocation; (2) detect whether the physiological responses are coordinated with the morphological results; (3) analyse the mechanism of drought resistance of *H. contortus*.

## Methods

### Study site

The experiment was conducted in Yuanmou station of the Institute of Mountain Hazards and Environment, Chinese Academy of Sciences, located in Yuanmou county (101.35–102.05°E, 25.25–26.07°N), Yunnan province, China, and belonged to the dry-hot valley region. The climate is subtropical with pronounced dry and wet seasons. The wet season lasts from June to October, during which time the precipitation is 623.95 mm, accounting for more than 90 % of the total rainfall. The dry season is very long and lasts up to 6–7 months (November to May). In the dry-hot season (February to May), water and heat are particularly unmatched.

### Experimental materials

Seeds of *H. contortus* were collected from the field and sown under natural conditions in May 2011. Seedlings with a similar size were transplanted individually into plastic pots (26 × 20 × 28 cm; upper diameter × lower diameter × height) in June 2011 and all plants were fully watered to ensure healthy growth. The aboveground tissues of *H. contortus* were withered and yellow in the winter, but re-greened in the dry-hot season. The pots were filled with 10 kg dry red soil (Luvisols in FAO soil taxonomy), which was widely distributed in Yuanmou. The contents of alkaline nitrogen, available phosphorus and available potassium in the experimental dry red soil were 31.5 ± 2.0, 1.3 ± 0.1 and 61.7 ± 0.8 (mg/kg), respectively. By measuring soil water characteristic curve, we found that the field moisture capacity and wilting coefficient (soil water potential was approximately −1.5 MPa) were 11.84 and 7.4 %, respectively. In our field survey, plants survived in the area when the soil water content was 2.45 %. Therefore, before the experiment was carried out, the permanent wilting coefficient of *H. contortus* was determined. *Heteropogon contortus* showing uniform growth were fully watered until water percolated from the bottom of the pots. Watering was then stopped and changes in the leaves were observed. When leaves began to wither, the plants were re-watered. The permanent wilting coefficient of *H. contortus* in this study was a range of soil water content, namely the corresponding soil water content when leaves either could or could not be recovered to the normal state after re-watering, which was 3.58–4.48 %.

### Experimental design

Before the drought-stress treatment in the dry-hot season, all plants were fully watered (until water percolated from the bottom of the pots). When seedlings regrew well, the drought-stress test was performed. According to the characters of dry red soil and the permanent wilting coefficient of *H. contortus*, seedlings were treated with four different soil water levels: >85 % (control, CK), 70–75 % (light stress, LS), 55–60 % (moderate stress, MS) and 35–40 % (severe stress, SS) of field capacity, respectively. In other words, the corresponding mass water content was >10.06 % (CK), 8.28–8.88 % (LS), 6.51–6.70 % (MS) and 4.14–4.74 % (SS), respectively. From the wilting coefficient of the dry red soil, we can infer that the soil water potential was greater than −1.5 MPa in LS treatment, and it was near to −1.5 MPa in MS treatment and far less than −1.5 MPa in SS treatment. Sixty seedlings with a similar height and crown size were used for each treatment with 15 replications. By natural drying, all seedlings reached the stress level on 15 April 2012. Then, the soil surface within the pots was covered with a plastic membrane to prevent soil evaporation. When plants were watered, the plastic membrane was uncovered carefully. Each pot was weighed and watered daily at 18:00 to ensure that its soil water content was consistent with the desired treatment value during the experiment.

**Fig. 1:**
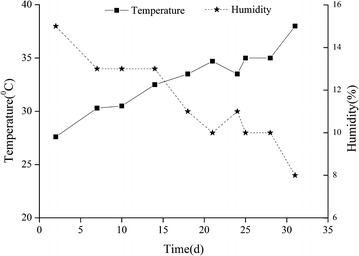
Temperature and relative humidity between 9:00 and 11:00 during the period of soil water stress

All pots were randomly arranged in a movable and ventilated awning to protect them from natural rainfall and were periodically rotated to minimise the effects of environmental heterogeneity, such as light and wind. Moreover, mean temperature and relative humidity between 9:00 and 11:00 were recorded using a thermo-hygrometer (WSB-2, China) during the experimental period (Fig. 1). The stress experiment ended on 13 May 2012, when leaves in the SS treatment were wilted and curled, which were the symptoms of permanent wilting. Subsequently, the remaining plants were re-watered adequately for 2 days to test their recovery.

### Observation and measurements

#### Growth characteristics

Growth was analysed at three stages during the experimental period: initial (plants experienced about 10 days of stress), middle (plants experienced about 20 days of stress) and late (plants experienced about 30 days of stress). Five plants per treatment were monitored to measure height and crown width (two vertical directions); then, the crown area was calculated by the formula of a circle (S = ∏r^2^) with the maximum crown width as the diameter. Five similar and well-expanded leaves from each treatment were transformed into digital pictures with a scanner and the leaf area (LA) was calculated from the pictures using ArcMap.

The relative growth rate (RGR) of each index was estimated using Eq. 1:

1 $${\text{RGR}} = {{\left( {\ln {\text{X}}_{2} { - }\ln {\text{X}}_{1} } \right)}/ {\left( {{\text{T}}_{2} { - }{\text{T}}_{ 1} } \right)}}$$

where X_1_ and X_2_ represent index values at the initial and late stages, respectively; T_1_ and T_2_ are measurement time.

#### Biomass and its distribution

Five seedlings from each treatment were harvested destructively at the initial, middle and late stages of the experiment. The roots were excavated carefully and cleaned with tap water. Shoots and roots were separated and dried at 85 °C to obtain a dry weight. The root mass ratio (RMR), shoot mass ratio (SMR) and root-to-shoot ratio (R/S) were calculated as:

2 $${\text{RMR}} = \left( {{{{\text{root}}\;{\text{biomass}}}/ {{\text{total}}\;{\text{biomass}}}}} \right) \times 100\;\%$$

3 $${\text{SMR}} = \left( {{{\text{shoot biomass}} / {\text{total biomass}}}} \right) \times 100\;\%$$

4 $${{\text{R}} / {\text{S}}} = \left( {{{\text{root biomass}} / {\text{shoot biomass}}}} \right) \times 100\;\% \,$$

#### Water retention

The tissue water content (WC) primarily includes free water (FW) and bound water (BW). FW has been identified with those water molecules that are not in direct contact with the solid, and BW to those that do come into direct contact (Velazquez et al. 2003). FW directly takes part in the physiological and biochemical metabolism in plants, while BW cannot do (Mao et al. 2012). FW, BW, WC and relative water content (RWC) were measured on days 3, 9, 15, 21 and 27 from the start of the experiment and after re-watering, following the methodology described by Ding et al. (2008). The FW was measured using an Abbe refractometer (WYA-2W, China). Several well-expanded leaves with uniform growth from each treatment were selected to separate into 150 small discs and were immediately placed into weighing bottles (40 × 25 mm) (50 slices per bottle randomly). After covering the bottle cap, they were weighed to obtain the fresh weight. Then, about 5 mL of 60–65 % sucrose solution was added to the bottle, which was placed in darkness for 4–6 h. The concentration of the sucrose solution was then determined using the Abbe refractometer. The FW was then calculated as:

5 $${\text{FW}}\left( \% \right) = \frac{{{\text{W}}_{1} \times \frac{{{\text{C}}_{1} - {\text{C}}_{2} }}{{{\text{C}}_{2} }}}}{{{\text{W}}_{2} }} \times 100\;\%$$

where W_1_ is the weight of the sugar solution (g); W_2_ is the fresh weight of leaves (g); C_1_ is the mass fraction of the sugar solution without leaves (%), and C_2_ is the mass fraction of the sugar solution with leaves (%).

The WC and RWC were measured by the oven-drying method. After measuring the fresh mass (FM), leaves were left in deionised water for 12 h to obtain the mass at full turgor (TM). Dry mass (DM) was obtained after drying the samples in an oven at 85 °C to constant weight. Then WC, BW and RWC were calculated as:

6 $${\text{WC}}\,\left( \% \right) = {{\left( {{\text{FM}}{ - }{\text{DM}}} \right)} / {\text{FM}}} \times 100\;\%$$

7 $${\text{BW}}\left( \% \right) = {\text{WC}} - {\text{FW}}$$

8 $${\text{RWC }}\left( \% \right) = {{\left( {{\text{FM}} - {\text{DM}}} \right)}/ {\left( {{\text{TM}} - {\text{DM}}} \right)}} \times 100\;\%$$

Leaf water potential (LWP) was measured on days 4, 9, 14, 19, 22 and 27 from the start of the experiment and after re-watering using a dewpoint potentiometer (PYSPRO, USA). Well-expanded leaves were sampled with a punch, and were placed into a C-52 sample chamber for 15 min.

#### Chlorophyll fluorescence

Chlorophyll fluorescence parameters were determined on days 3, 6, 11, 15, 19, 24 and 29 of the experiment and after re-watering using a chlorophyll fluorescence spectrometer (Handy PEA 1024, Germany). Before measurement, mature, fully developed leaves from the lower part of the rosette (Jung 2004) were nipped and were adapted to darkness for 20–30 min. Using solar energy as a photochemical energy, measurements were taken on three mature leaves from three pots for each treatment. The minimum fluorescence (F_0_) and maximal fluorescence (Fm) of dark-adapted leaves of photosystem II (PSII) were assessed concurrently. The maximum quantum efficiency (Fv/Fm) of PSII photochemistry was determined as:

9 $${{\text{Fv}} /{\text{Fm}}} = {{\left( {{\text{Fm}} - {\text{F}}_{0} } \right)}/ {\text{Fm}}}. \,$$

### Data analysis

Statistical tests were performed with SPSS 16.0. Data were expressed as mean ± standard error (SE). Differences in growth parameters, biomass, water-content parameters, LWP, and chlorophyll fluorescence parameters were tested using one-way analysis of variance (ANOVA), and compared using the least significant difference method (LSD). In addition, correlation analysis was used to analyse the relationship between LWP and environmental variables.

## Results

### Growth characteristics

**Fig. 2:**
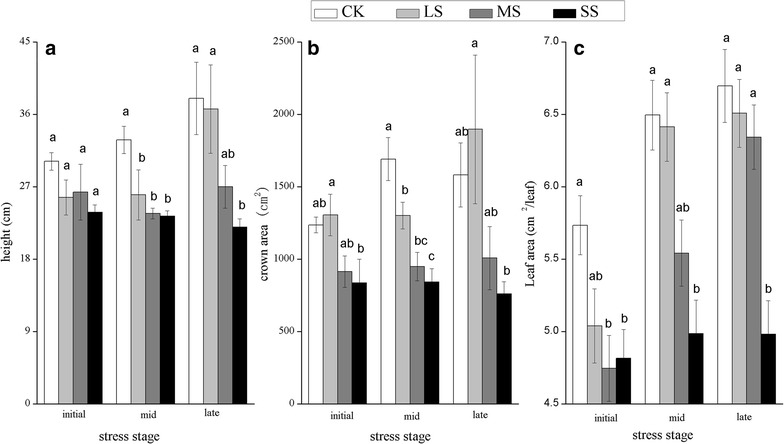
Plant height (**a**), crown area (**b**) and leaf area per leaf (**c**) of *Heteropogon contortus* at different stages under four water levels. Values represent means (n = 5) with standard* error bars*. The *different letters* above the* bars* indicate significant differences among the treatments at the same stage at P < 0.05

Drought treatment caused a decrease in plant height, crown area and LA in *H. contortus*, in the order CK > LS > MS > SS (Fig. 2). At the late stage, only the SS level was significantly lower than that of CK (*P* < 0.05), was about 42.11, 52.02 and 25.6 % lower than CK for plant height, crown area and LA, respectively. The RGR of plant height, crown area and LA in LS was highest, up to 0.018, 0.019 and 0.013, respectively, whereas that in the SS treatment showed a negative growth, which was −0.004, −0.005 and 0.002 for plant height, crown area and LA, respectively. Furthermore, the number of leaves was significantly different among treatments at the late stage and was 130 ± 5.48, 115 ± 7.06, 85 ± 3.28 and 69 ± 5.86 for CK, LS, MS and SS, respectively, and some leaves under SS were curly and folded and showed a wilted symptom.

### Biomass and its distribution characteristics

**Fig. 3:**
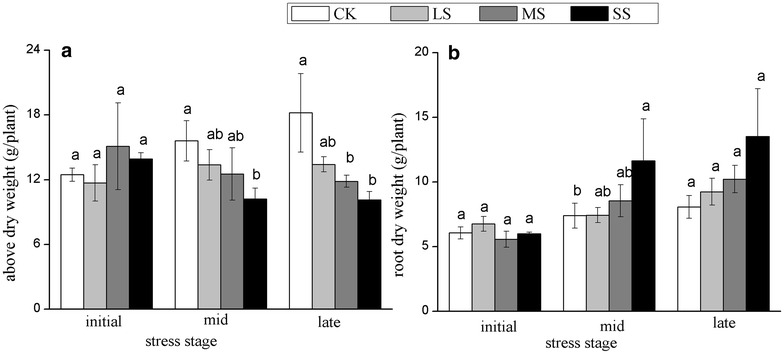
Aboveground biomass (**a**) and root biomass (**b**) of *Heteropogon contortus* at different stages under four water levels. Values represent the means (n = 5) with standard* error bars*. The* different letters* above the* bars* indicate significant differences among the treatments at the same stage at P < 0.05

With the increasing intensity and duration of water stress, aboveground biomass decreased gradually (Fig. 3a), whereas root biomass increased (Fig. 3b). Root biomass in the SS treatment was 67.49 % more than that in CK. The drought treatments and duration caused a decrease in the SMR and an increase in the RMR, thus resulting in an increased root/shoot (R/S) ratio (Table 1). At the late stage, the ratio of R/S in the SS treatment was about two times higher than that in CK (*P* < 0.05).

**Table 1:** Shoot mass ratio (SMR), root mass ratio (RMR) and root/shoot ratio (R/S) of *Heteropogon contortus* at different stages under four different water levels

Levels	Initial stage			Mid stage			Late stage		
	SMR	RMR	R/S	SMR	RMR	R/S	SMR	RMR	R/S
CK	67.33 ± 1.09a	32.67 ± 1.09a	48.60 ± 2.38a	64.84 ± 1.3a	35.14 ± 1.3c	54.35 ± 3.00c	68.25 ± 2.35a	31.75 ± 2.35c	47.03 ± 4.89b
LS	63.43 ± 3.59a	36.57 ± 3.59a	59.17 ± 6.88a	56 ± 2.54c	44 ± 2.54a	79.71 ± 8.41a	59.55 ± 1.7ab	40.45 ± 1.7bc	68.34 ± 4.83b
MS	71.22 ± 4.75a	28.78 ± 4.75a	41.76 ± 10.14a	62.27 ± 1.83ab	37.73 ± 1.83bc	61.00 ± 4.69bc	53.95 ± 1.74bc	46.05 ± 1.74ab	85.96 ± 6.27ab
SS	69.87 ± 0.54a	30.13 ± 0.54a	43.15 ± 1.11a	57.69 ± 0.73bc	42.31 ± 0.73ab	73.44 ± 2.22ab	45.56 ± 5c	54.44 ± 5a	127.90 ± 25.65a

Values are means ± standard errors of five replicates. Different lowercase letters in each column indicate significant difference among treatments at P ≤ 0.05

### Water retention

**Fig. 4:**
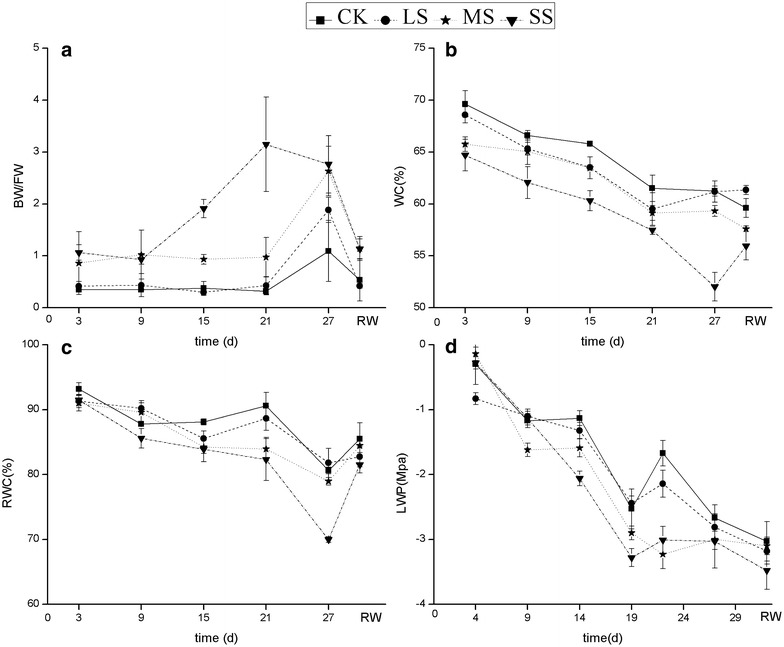
Bound water/free water ratio (**a**), total water content (**b**), relative water content (**c**) and leaf water potential (**d**) of *Heteropogon contortus* on different days under four different water levels. Values represent the mean ± standard error (n = 3) and RW represents the day after re-watering

Soil water stress caused an increase in BW/FW and a decrease in WC, RWC and LWP (Fig. 4). During the progression of water stress, FW decreased gradually, whereas BW increased, thus causing a subsequent increase in the BW/FW ratio (Fig. 4a), and it was significantly higher in SS treatment than that in CK finally (*P* < 0.05). The RWC was high all the time, and was nearly 70 % in the SS treatment during the late period, although it was significantly lower than that in CK (*P* < 0.05). No significant differences were observed in LWP (*P* = 0.781) among treatments. There were highly significant correlations between LWP and weather factors (Table 2). The LWP was negatively correlated with temperature and was positively correlated with relative humidity. At the end of the stress treatment, the percentage change in WC, RWC and LWP under drought conditions (including LS, MS and SS) relative to CK was small and was 0.08–15.05, 1.45–13.20 and 5.24–13.48 %, respectively. However, the magnitude of the variation in BW/FW was greater (72.26–152.83 %) under the stressed conditions compared to under the CK treatment. After re-watering for 2 days, the BW/FW ratio decreased to the initial value, WC and RWC increased slightly, but LWP continued to decrease.

**Table 2:** Pearson’s correlation coefficients (r) and their level of significance (P) showing the relationships between LWP and weather factors

Levels	Temperature		Relative humidity	
	r	P	r	P
CK	−0.859*	0.013	0.861*	0.013
LS	−0.934**	0.002	0.942**	0.002
MS	−0.832*	0.020	0.820*	0.024
SS	−0.884**	0.008	0.832*	0.020

*, ** indicate the significance at the P < 0.05 and P < 0.01 level, respectively

### Chlorophyll fluorescence

**Fig. 5:**
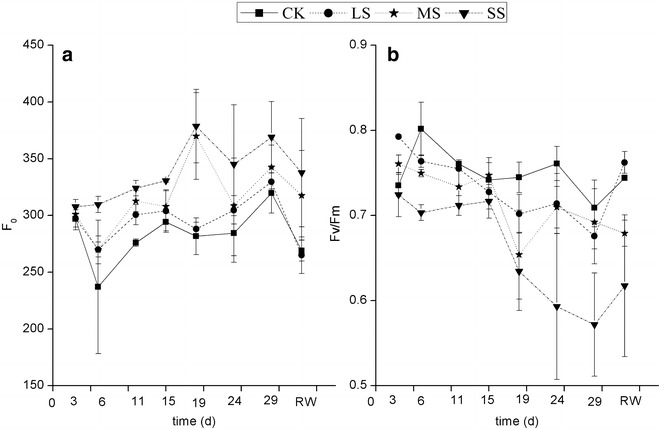
F_0_ (**a**) and Fv/Fm (**b**) of *Heteropogon contortus* on different days under four different water levels. Values represent the mean ± standard error (n = 3) and RW represents the day after re-watering

During the whole stress period, F_0_ increased and the Fv/Fm ratio decreased gradually (Fig. 5). At the end of the stress treatment, F_0_ in the SS treatment increased by 19.93 % and the Fv/Fm ratio decreased by 21.07 %, compared to the initial value. In addition, at the end of the stress treatment, the Fv/Fm ratio decreased to below 0.6, which was significantly lower than that in the CK treatment (*P* < 0.05). Two days after re-watering, the F_0_ value of all stress levels decreased, the Fv/Fm ratio increased slightly, but was still less than 0.7 in the SS treatment.

## Discussion

### Responses of morphological traits of *H. contortus* to drought

Continuous water stress results in fewer and smaller leaves and shorter plants, which retain water in their leaf tissues (Haffani et al. 2014). The LA showed significant differences at the initial stage, which were more rapid than the observed differences in plant height. Because of the high sensitivity of foliar expansion to water stress, the inhibition of leaf growth is the first response to water deficit (Du et al. 2010). Due to the rapid reduction in LA, the reduction in crown area was also observed at the initial stage (Fig. 2).

The LSD showed that only height, crown area and LA in the SS treatment was significantly different from those in the CK treatment at the late stage, indicating the high ability of *H. contortus* to resist drought. Furthermore, at the late stage, some leaves in the SS treatment were folded or twisted. This is common in grasses and contributes to the low rate of soil water demand (Guenni et al. 2004). In addition, *H. contortus* showed a higher RGR of height, crown area and LA in the LS treatment than in other treatments, showing that mild water stress can promote the growth of *H. contortus*. A higher growth rate in mild water stress conditions has been observed in plants such as *Carapa guianensis* (Azevedo 2013). However, because of the negative RGR, severe water stress inhibited the growth of *H. contortus*, which is consistent with the LSD.

In conditions of drought, growth inhibition eventually results in a reduction in aboveground biomass and plants allocate more biomass to roots to increase water uptake after soil drying (Xu et al. 2006). The mechanism of this redistribution is believed to be associated with the accumulation of abscisic acid and a reduction in the cytokinin level (Kudoyarova et al. 2013). However, in drought conditions (including LS, MS and SS), the aboveground biomass of *H. contortus* decreased and the root biomass simultaneously increased (by 14.55–67.49 %), compared to in well-watered conditions, thus leading to a distinct high R/S ratio (Fig. 3; Table 1). The increase in root biomass contrasts with findings for other plants (Fernandez and Reynolds 2000; Guenni et al. 2002; Blum 2005; Bahrani et al. 2010; Haffani et al. 2014) and other studies on *H. contortus* (Williams and Black 1994), where root dry weight decreased under drought treatment. Van Den Berg and Zeng (2006) reported that *H. contortus* in South Africa involves a dramatic increase in root length under slight drought stress, it is an adaptive response to reach deeper water in the soil. Similarly, the massive root biomass in this study is also an adaptive response to reach more water. Avoidant species usually maximise water uptake by increasing root mass and reducing the leaf transpiration area (Chaves et al. 2003). Therefore, it is reasonable to conclude that *H. contortus* tolerates drought via an avoidance mechanism.

### Responses of physiological characteristics of *H. contortus* to drought and re-watering

With the progression of stress, the BW/FW increased, and WC, RWC and LWP decreased (Fig. 4). This might be partly due to the weather, which became hotter and drier (Fig. 1). When the soil moisture and the relative humidity are low and the ambient temperature is high, drought stress occurs (Lipiec et al. 2013). Heat and drought stresses often occur concomitantly, especially in dry-hot valleys. This combination of multiple stresses can have a great adverse impact on plants (Zhang et al. 2010); high temperature strongly affects water retentions when water is limiting (Machado and Paulsen 2001). Therefore, *H. contortus* constantly adjusts their water content to counteract the increased drought stress.

The BW/FW ratio was the most sensitive parameter to drought; it increased rapidly after the ninth day of severe stress, and decreased to the initial value after re-watering. Compared with four desert species and a semi-arid species, whose BW/FW was 0.45–1.58 (Ma et al. 2008), the BW/FW ratio of *H. contortus* ranged from 0.32 to 3.15. At the end of the stress treatment, the BW/FW ratio under drought conditions was 72.26–152.83 % more than in the CK treatment. The FW of a plant restricts metabolic intensity, whereas BW is closely related to the resistance of a plant (Sun et al. 2005). A relatively low FW, high BW and a high ratio of BW/FW favours water retention, enhances plant resistance to stress, and thereby enables a species to adapt to an arid environment (Ma et al. 2008). At the same time, when BW/FW is high, the metabolism of a plant is restricted, thus causing negative RGR of *H. contortus* under SS.

Consistent with Ashraf and Yasmin (1995), LWP was not affected by drought. According to the theory of the soil–plant–atmosphere continuum system, LWP is influenced by soil and the atmosphere. In agreement with Rodriguez et al. (2004), this study showed that LWP was negatively correlated with air temperature, but was positively correlated with relative humidity (Table 2). This relationship might explain the non-significant differences among water-stress levels in LWP, which is more affected by the weather. Therefore, LWP still decreased after re-watering, and the recovery of LWP may be delayed. Compared with other grasses in the Loess hilly gully region on Loess Plateau in China (Xu et al. 2006), *H. contortus* can maintain a higher RWC (about 70 %) even at a lower LWP under SS (<−3 MPa), implying that *H. contortus* is more tolerant to drought (Haffani et al. 2014). The high RWC might be due to the reduction of LA, the extensive root system, and the increased BW. Besides, drought will reduce the osmotic potential of *H. contortus* (Williams and Black 1994). The lower osmotic potential favours the conservation of water in the tissue, and the reduction is mainly due to dehydration or the active accumulation of solutes (Chartzoulakis et al. 2002). It has been reported that the osmotic adjustment of *H. contortus* to water stress, mainly through accumulation of solutes and partly through the variation in the proportion of BW (Wilson et al. 1980). In our study, the FW content decreased gradually and BW increased with the stress going. Therefore, one the one hand, the BW is beneficial to water retention as we mentioned above; on the other hand, the increased BW proportion may reduce the osmotic potential and save water, thus maintaining a high RWC. The observed high RWC at a low LWP demonstrates that *H. contortus* resists drought by dehydration avoidance, a way of drought tolerance (Kozlowski and Pallardy 2002). Osmotic adjustment is a significant strategy for plant drought tolerance, and it may improve root growth as water deficits become severe (Fang and Xiong 2015). Both the accumulation of solutes and the increase of BW are responsible for the osmotic adjustment of *H. contortus*, but which is more important in our study requests further research.

Notably, during the measurements of leaf water content of *H. contortus*, the leaves did not sink easily in the water, showing hydrophobicity. It can be speculated that the cuticles of *H. contortus* leaves might possess a wax layer (Müller and Riederer 2005). Many herbaceous species in arid environments possess a wax layer, which plays a crucial role in minimising water loss (Ashraf and Yasmin 1995; Bolger et al. 2005; Zhou et al. 2014). Therefore, in conditions of water stress, the root mass of *H. contortus* increases to absorb more water from deep soil, to supply the growing parts of the leaves. In addition, the wax layer on the leaves reduces leaf transpiration and maintains water in the leaves to protect *H. contortus* against drought in the field, thus making a high RWC.

In the absence of environmental stress conditions, Fv/Fm (after being dark-adapted) is relatively stable, with a value of about 0.75–0.85 in general (Siam et al. 2008; Guo et al. 2008). Some seedlings have a value of less than 0.75, but greater than 0.7 (Galle and Feller 2007). The present study showed that the Fv/Fm value of CK, LS and MS was above 0.7, but was only 0.572 under SS during the late period (Fig. 5b), indicating a decrease in the maximum photochemical yield of PSII. Mild drought stress does not affect the maximum efficiency of PSII primary photochemistry, which is only affected under severe drought conditions (Baker and Rosenqvist 2004; Pukacki and Kaminska-Rozek 2005; Boughalleb and Hajlaoui 2011). Similarly, we found that only severe water stress affected the Fv/Fm of *H. contortus*, which was consistent with the growth characteristics.

After re-watering for 2 days, F_0_ decreased and Fv/Fm increased, but Fv/Fm under SS remained lower than 0.7. The down-regulation of Fv/Fm during the stress was at least partially reversible after re-watering, indicating the continued functionality of the photosynthetic apparatus during severe stress. If the decrease in Fv/Fm can be explained by an increase in F_0_, then the PSII is not functional (Figueroa et al. 1997). The current study showed that the decrease in Fv/Fm, which was associated with a rise in F_0_ (Fig. 5), indicates the occurrence of photodamage. Resco et al. (2008) showed that after 2 months of drought, the Fv/Fm ratio of *H. contortus* can be recovered from 0.3 to the normal level (0.8) on the third day after irrigation, showing that although photosynthesis is inhibited, *H. contortus* has the ability to recover. Ripley et al. (2010) also reported that C_4_ species (including *H. contortus*) are metabolically more sensitive to drought than C_3_ species and recover more slowly from drought. Therefore, the reduction in Fv/Fm might represent the reversible photo-inactivation of PSII centres to adapt to water stress. Leaf rolling (Nar et al. 2009), leaf epicuticular wax (Robinson et al. 1993; Mohammadian et al. 2007) and reversible photo-inactivation (Feng et al. 2002) might protect PSII functionality from damage induced by drought stress. Moreover, this further demonstrates the potential existence of leaf epicuticular wax on *H. contortus*.

## Conclusions

Leaf rolling, wax accumulation and a well-developed root system are mechanisms of drought avoidance (Fang and Xiong 2015). By combining many types of avoidance mechanisms and some tolerant mechanisms, *H. contortus* is resistant to drought stress. Like most grasses, the strong drought resistance can attribute to the following three reasons. First, by reducing transpiration and retaining water, such as the smaller LA, the slower RGR, and the increased BW; second, by extracting more water, such as the extensive root mass; third, by maintaining normal physiological activities, such as PSII functionality. But differing from other grasses, the increases in BW/FW and R/S ratios are important strategies in the response to soil water stress. Morphological and physiological responses show that only severe drought stress inhibits the growth of *H. contortus* and negatively affects its physiologic adjustment. In the process of vegetation restoration, mild water stress is tolerated, and a water supply above 40 % FC of dry red soil is recommended to maintain vigorous seedling growth.

## Abbreviations

CK: control
LS: light stress
MS: moderate stress
SS: severe stress
LA: leaf area
RGR: relative growth rate
RMR: root mass ratio
SMR: shoot mass ratio
R/S: root-to-shoot ratio
BW/FW: bound water/free water
RWC: leaf relative water content
LWP: leaf water potential
Fv/Fm: variable fluorescence/maximum fluorescence, the maximum quantum efficiency
